# Immunohistochemical Expression of CDX2 in Gastric Carcinoma

**DOI:** 10.30699/IJP.2022.530631.2648

**Published:** 2022-03-08

**Authors:** Ahmed Avan Sardar, Jalal Ali Jalal, Kalthuma Salih Hamad Ameen

**Affiliations:** 1 Department of Basic Sciences/Pathology, College of Medicine, Hawler Medical University, Erbil, Iraq.; 2 Department of Histopathology, Pathology Lab, Rizgary Teaching Hospital, Erbil, Iraq

**Keywords:** CDX2, Gastric carcinoma, Immunohistochemistry

## Abstract

**Background & Objective:**

Gastric cancer (GC) persists to be a major health issue globally, and the need to investigate new molecular markers for improving the survival of patients continues. CDX2 is a homeobox caudal protein family member encoded by the *CDX2* gene and is probably playing a role in intestinal epithelial differentiation and proliferation. This study aimed to assess the expression of this protein in gastric cancer cells in addition to its correlation with multiple clinicopathological parameters.

**Methods:**

This observational retrospective study was carried out on 80 gastric cancer cases in Erbil, Iraq. CDX2 protein immunoexpression in tumor cells, as well as its correlation with several clinicopathological criteria, were investigated.

**Results:**

CDX2 was detected in 38.75% of GC patients. We found a significant correlation between *CDX2* expression and the age of patients (*P*=0.02). Even though the protein was more expressed in tumors with negative lymphovascular invasion and intestinal GC, there was no significant correlation between the expression of this protein and invasion. In addition, CDX2 expression was not significantly correlated with patient gender, tumor grade, nodal status, and tumor stage.

**Conclusion:**

CDX2 expression was observed to be downregulated in younger patients. It could be due to the higher frequency of diffuse GC, in which CDX2 is expressed less than the intestinal type, in younger individuals.

## Introduction

Gastric cancer (GC) is one of the most prevalent cancers and is currently among the top third major causes of death due to malignancy at the global level. It stands at the fifth order of most commonly diagnosed cancers and accounts for nearly 10% of total mortalities worldwide every year, with the rate being higher in developing countries. The highest incidence rates are in Eastern Asia (1). Regarding the Kurdistan Region of Iraq, GC lies at the eighth order among most common cancers (2). 

 Advanced GC persists in having a poor prognosis despite therapeutic advancements, including surgery, chemotherapy, and radiotherapy. Although the detailed mechanisms of gastric carcinogenesis are not yet fully understood, several associated environmental and genetic factors have been reported to play an important role in promoting GC, such as *Helicobacter pylori* infection and mutation in the *E-cadherin (CDH1)* gene (3). 

It has been epidemiologically exhibited that GC does not appear in the normal epithelial lining of the stomach de novo. What most accept as a concept of GC progression is that most cases evolve through a multistep process initiating with superficial gastritis, progressing to atrophy with intestinal metaplasia followed by the development of dysplasia, and finally carcinoma (4). The GC is histologically sorted as either intestinal or diffuse types by the Lauren classification system (5).

It is essential to perceive the molecular processes of the pathogenesis of GC to establish novel therapeutic approaches for improving patient outcomes. Therefore, it is necessary to identify beneficial molecular markers to estimate malignancy potential. The CDX2, a transcription factor, belongs to the caudal-related homeobox gene family. It is encoded by the *CDX2* gene and is important in regulating the proliferation and differentiation of intestinal cells and maintaining the intestinal phenotype (6). Claudin-2, LI-cadherin, and Desmocollin-2, which play role in cell-cell adhesion, are some of the transcriptional targets of CDX2 (7-9). *CDX2* gene is found on human chromosome 13q12–13 (10). The expression of this gene is detected specifically in the small intestine, colon, and intestinal metaplasia of the stomach but not in normal adult esophageal and gastric epithelial tissues (11). Furthermore, it participates in bringing on polarity and columnar phenotype (12). Mucosal epithelial cell metaplasia could arise from ectopic *CDX2* in gastric mucosa, one of the early steps in gastric carcinogenesis (13, 14). CDX2 protein regulation remains to be clearly defined in the GC cell line. However, studies have demonstrated the participation of the bone morphogenetic protein (BMP) signaling pathway as a crucial event in the process, where proteins of the pathway are highly expressed in cells infected with *H. pylori*. Moreover, BMP2/BMP4 distinctively has been shown to elevate the expression of CDX2 protein through signaling SMAD family member 4, also known as SMAD4 (15, 16). On the other hand, a Sry-related HMG box protein, also known as SOX2, is proposed to be a repressor of CDX2 expression (17).

Our study aimed to assess the expression of CDX2 in gastric adenocarcinoma by immunohistochemistry in addition to investigating the relationship between CDX2 expression and some clinicopathological parameters, such as the age and gender of patients, as well as tumor type, tumor grade, lymphovascular invasion, lymph node status, and tumor stage.

## Material and Methods

Eighty formalin-fixed, paraffin-embedded blocks of gastrectomy specimens diagnosed as GC during January 2018-January 2020 were obtained non-randomly from the ﬁles of the histopathology laboratory of Rizgary Teaching Hospital and a private histopathology lab in Erbil. Two sections were taken from each block, one stained with H&E for histological analysis and the other consumed for immunohisto-chemical evaluation regarding CDX2 expression. The histological grade is coded as Well-moderately differentiated and poorly differentiated, including signet ring carcinoma (18). According to the American Joint Committee on Cancer and the Union for International Cancer Control (UICC), pathological tumor staging is performed by grouping the various TNM components (19). We assessed CDX2 expression in GC cases using the immunohistochemical method. 

Ethical approval was obtained from the Ethics Committee of Kurdistan Board for Medical Specialties. Formal written informed consent was not required with a waiver by the Research Ethics Committee of Kurdistan Board for Medical Specialties (21/9/2020, No.628).

**Immunohistochemistry**

IHC staining was performed using the envision-labeled peroxidase system (Dako). In brief, two sections of 4 μm thickness were prepared from each block, one to be used for H&E examination and the other used for immunohistochemical analysis. Sections were deparaffinized, rehydrated through a graded ethanol series, and incubated for 10 min in 3% H_2_O_2_ to inhibit endogenous hydrogen peroxidase activity. Antigen retrieval was carried out by autoclaving in citrate buffer (pH=6) for 10 min. This was followed by applying a primary antibody of CDX2 at 4°C overnight. After rinsing in phosphate buffer saline, sections were treated with a peroxidase-labeled polymer attached to gout antimouse immunoglobulin as a secondary antibody (Dako) for 30 min at 37°C followed by counterstaining with hematoxylin. The slides were examined by an Olympus CX23 light microscope by two pathologists blindly and without having prior information about the clinical data of the cases. Normal colonic mucosal tissue was used as the positive control. For negative controls, the primary antibody was omitted in each run. According to the literature, CDX2 expression is mostly found in cell nuclei. In the present study, only nuclear staining was regarded as positive (20, 21). For scoring purposes, the cut-off value for antibody reactivity in the tumor cells was set at 10%, likewise considered convenient in several other studies (21-23). Photomicrographs were captured by a Canon EOS 750D camera at ×400.

**Statistical Analysis**

Statistical analysis was performed by the SPSS software version 23, and the level of significance was considered P-value≤0.05. The Chi-square test was used to evaluate the correlation between CDX2 expression and clinicopathological parameters**.**

## Results

**Fig. 1:**
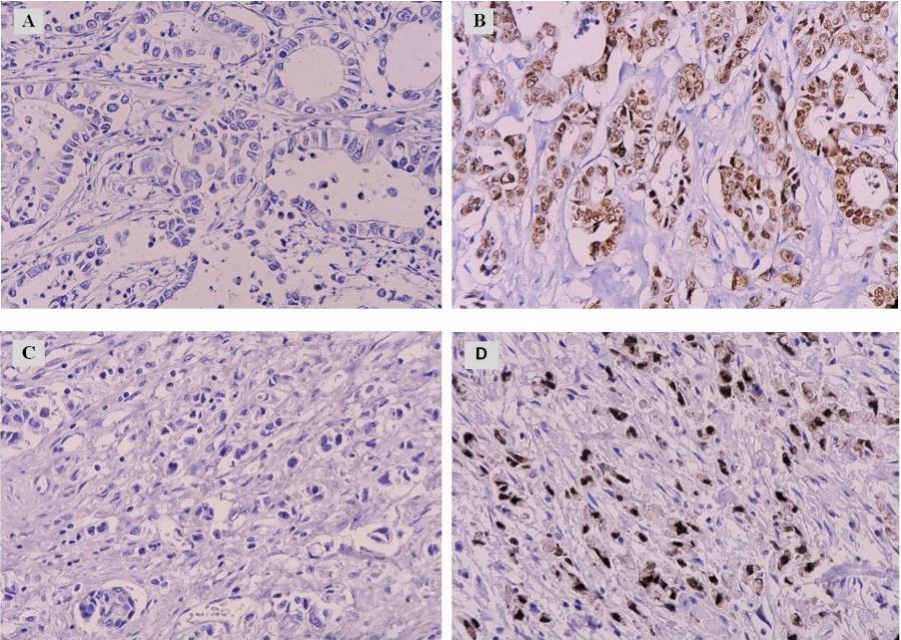
Immunohistochemical evaluation of CDX2 expression in GC cases at ×400 magnification. Cases of intestinal (A) and diffuse (C) GC demonstrate an absence of CDX2 expression in the tumor cell nuclei. Nuclear expression of CDX2 could be observed in intestinal (B) and diffuse GC cells (D)

In the current study, CDX2 expression in 80 GC patients was assessed and associated with some clinicopathologic parameters. Sections were evaluated for nuclear CDX2 expression (Figure 1). CDX2 protein was expressed in 38.75% (31/80) of the cases, while a larger proportion of 61.25% (49/80) was negative for the protein. We found that 55% (44/80) of the cases were male, and 45% (36/80) were female, with a male to female ratio of 1.2:1. Further details of the clinicopathological data are shown in (Table 1). 

We observed a significant correlation between the age of patients and CDX2 expression in tumor cells (*P*=0.02). CDX2 was expressed only in 12.5% (2/16) of the patients aged 50 years and younger, while 87.5% (14/16) of that age group were negative for the protein. The majority of cases were positive for lympho-vascular invasion, had lymph node metastasis, and were of high tumor stage represented by 80% (64/80), 86.3% (69/80), and 70% (56/80), respectively. Additional details are outlined in Table 2.

**Table 1:** Clinical and demographic data of the patients diagnosed as GC

Variables	Categories	Number	Percent
Gender	Male	44	**55**
	Female	36	**45**
Age groups	≤50 years	16	**20**
	>50 years	64	**80**
Tumor type	Intestinal type	48	**60**
	Diffuse type	32	**40**
Tumor grade	Well-moderately differentiated	33	**41.3**
	Poorly differentiated	47	**58.7**
lymphovascular invasion	Positive	64	**80**
	Negative	16	**20**
Nodal status	Positive	69	**86.3**
	Negative	11	**13.8**
Tumor stage	1-2	24	**30**
	3-4	56	**70**
CDX2 score	Negative	49	**61.25**
	Positive	31	**38.75**
Total		**80**	**100**

**Table 2:** Correlation between CDX2 expression and the clinicopathological parameters

Variables	Categories	CDX2 score		P-value
		Negative	Positive	
Gender	Male	25(54.3%)	19(55.9%)	**0.892**
	Female	21(45.7%)	15(44.1%)	
Age groups	≤50 years	14(28.6%)	2(6.5%)	**0.02**
	>50 years	35(71.4%)	29(93.5%)	
Tumor type	Intestinal type	27(58.7%)	21(61.8%)	**0.782**
	Diffuse type	19(41.3%)	13(38.2%)	
Tumor grade	Well-moderately differentiated	21(45.7%)	12(35.3%)	**0.352**
	Poorly differentiated	25(54.3%)	22(64.7%)	
lymphovascular invasion	Positive	38(82.6%)	26(76.5%)	**0.497**
	Negative	8(17.4%)	8(23.5%)	
Nodal status	Positive	39(84.8%)	30(88.2%)	**0.751**
	Negative	7(15.2%)	4(11.8%)	
Tumor stage	1-2	13(28.3%)	11(32.4%)	**0.309**
	3-4	33(71.7%)	23(67.6%)	
Total		**46(100%)**	**34(100%)**	

## Discussion

The correlation we detected in this study is also recorded by Schildberg* et al.* (3) and Ha Kim* et al.* (24), who similarly perceived the reduced expression of CDX2 in the younger patient population compared to older patients. Moreover, Park* et al.* (20) stated more frequent expression of CDX2 in patients with dysplas-tic gastric epithelium, a change known for being one of the consequent steps of gastric carcinogenesis. This outcome could be explained based on the fact that GCs are much less common in young individuals. However, when present, they are more likely to be diffuse with lower expression of CDX2 than the intestinal type, as claimed by several studies (11, 20). 

CDX2 expression was more detected in the intestinal than the diffuse subtype regarding histolo-gical subtype. However, the difference was not statistically significant. On the other hand, some other investigations reported a significant relationship in this regard (25, 26). Furthermore, Song* et al.* (27) assumed that intra-epithelial tight junctions in GC cells were disruptted due to claudin-2 overexpression via CDX2 driven by CagA, which is encoded* H. pylori* leading to a less differentiated phenotype of the cells. However, controversial results can be attributed to the divergence of samples and staining procedures. Similar to Zhang* et al.* (28), we observed no significant correlation between CDX2 expression and tumor grade. 

It is noteworthy that we observed a non-significant negative relationship between lymphovascular inva-sion and CDX2 expression (*P*=0.5), which is in line with the findings of Ha Kim* et al.* (24). This may be explained by several studies showing the tumor-suppressive role of CDX2 protein (18, 25, 29). Contrary to the hypothesized negative correlation between nodal status and CDX2 expression (24), our data suggest no remarkable relationship in this particular aspect. This can also be observed in a study performed by Camilo* et al.* (30). This non-identical outcome may be attributed to dissimilarity in the sample size of studies. Furthermore, we found no significant correlation between CDX2 expression and the gender of patients, which is consistent with some other studies (22, 28, 31). However, some claimed a significant relationship between gender and CDX2 expression, demonstrating that CDX2 expression is more common among male patients (25). 

Furthermore, CDX2 is expressed in 45% (11/24) of low-stage and 41% (23/56) of high-stage tumors. How-ever, the correlation was not statistically significant, which is in line with the study performed by Tavga* et al.* (32). Others reported a significant correlation in this regard (20). CDX2 has been noted to play a role in assisting GC cells in migrating and invading (27, 33). Variations in the number of cases, staining techniques, and interpretations are among the possible reasons for these non-identical and inconsistent results. 

## Conclusion

GC is a recognizably heterogenic tumor characterized by distinct survival rates among different geographic locations. Moreover, the area is up to the moment widely debatable as witnessed by the extensive studies in the field with dissimilar and even conflicting results. Collectively, our data suggest a downregulation of CDX2 in the younger patient population. We believe that the latter point could serve as a beneficial finding in prognosis. However, to be more precise, further investigations might be required in this regard with larger sample size. All the contradictory views explain that still too little is known about the various regulatory roles of CDX2 in GC cells.

## Conflict of Interest

The authors declared no conflict of interest.

## Funding

The authors received no financial support for the research, authorship, and/or publication of this article.
